# The Role of High-Flow Nasal Cannula (HFNC) During Flexible Bronchoscopy in Adult Patients with Moderate Respiratory Dysfunctions: An Observational Study

**DOI:** 10.3390/jcm15020459

**Published:** 2026-01-07

**Authors:** Francesco Coppolino, Pasquale Sansone, Gianluigi Cosenza, Simona Brunetti, Francesca Piccialli, Marco Fiore, Clelia Esposito, Maria Caterina Pace, Vincenzo Pota

**Affiliations:** 1 Department of Woman, Child, General and Specialistic Surgery, University of Campania “L. Vanvitelli”, 80138 Naples, Italy; 2 Critical Area Department, Anesthesia and Intensive Care, AO Dei Colli, Vincenzo Monaldi Hospital, 80131 Naples, Italy

**Keywords:** ICU, flexible bronchoscopy, HFNC, respiratory failure

## Abstract

**Background/Objectives**: Flexible bronchoscopy (FB) enables airway exploration and diagnosis of various respiratory pathologies, but the sedation and instrumentation required during the procedure raise oxygen demand while reducing ventilation, which can lead to hypoxemia. Conventional oxygen therapy (COT) may not adequately prevent desaturations in high-risk groups, as patients with moderate respiratory deficiency. High-flow nasal cannula (HFNC) can deliver heated, humidified oxygen at high flow rates, generating low-level positive airway pressure, improving oxygenation, reducing dead-space, and enhancing procedure tolerance. Prior studies have shown that HFNC can improve gas exchange and reduce desaturations during bronchoscopy. However, evidence remains limited for patients with moderate respiratory deficiency, who are particularly vulnerable. Evaluating the feasibility and safety of HFNC in this population is essential to guide safe procedural practice. **Methods**: A retrospective observational study including patients undergoing FB with HFNC support between January and May 2025. Inclusion criteria were BMI between 18 and 30; age > 18 years old; moderate respiratory dysfunction, defined by pulse oximetry, Pulmonary Functional Tests (PFTs) and Arterial Blood Gas (ABG) analysis. Exclusion criteria were intolerance/contraindication to HFNC. Procedures were performed under basic monitoring. Primary outcome was occurrence of severe hypoxemia (SpO2 < 90%). Secondary outcomes were needed for rescue maneuvers, interruption for conversion to other ventilatory strategies, and hemodynamic instability. **Results**: No severe desaturations were recorded, all procedures were completed without rescue maneuvers or other ventilatory strategies, and no hypoxemia occurred. Mean duration of the procedure was 9 min. Vital parameters were maintained within the normal ranges, with a mean SpO_2_ during bronchoscopy of 98%. **Conclusions**: HFNC enables oxygenation and ventilation without adverse events in sedations for FB in patients with moderate respiratory deficiency.

## 1. Introduction

Flexible bronchoscopy (FB) involves the insertion of a flexible, fiber-optic bronchoscope through the oral or nasal route for direct visualization of the tracheobronchial tree. It has long been established as an essential diagnostic and therapeutic tool in respiratory medicine and critical care, allowing inspection of the airways and diagnosis of various respiratory pathologies, enabling collection of bronchial brushings, bronchoalveolar lavage (BAL), and tissue biopsies. Furthermore, it enables direct intervention for airway stenting, secretion removal, or foreign body retrieval [1]. Given the potential for discomfort, cough reflex activation, and procedural anxiety, sedation is generally required to enhance patient tolerance, facilitate procedural success, and ensure safety. Sedative agents such as propofol or midazolam are commonly utilized to achieve an appropriate depth of sedation, while maintaining spontaneous ventilation whenever possible [2,3].

FB is associated with a spectrum of potential complications, notably respiratory and hemodynamic disturbances. Among these, hypoxemia is particularly prevalent, with reported incidences ranging from 30% to 70%, and is often accompanied by a reduction in arterial oxygen tension (PaO_2_) of approximately 20 mmHg, especially during BAL, when large volumes of fluid are instilled into the distal airways [4]. Other commonly observed adverse events include cough, bronchospasm, dyspnea [5], hypercapnia, blood pressure fluctuations, and arrhythmias [6] directly correlated to the level of hypoxia [7]. These complications are particularly significant in patients with underlying pulmonary disease or compromised respiratory function, where even brief episodes of hypoxemia can precipitate clinical deterioration.

Several studies highlighted the need for supplemental oxygen administration during FB [8,9], yet the optimal delivery method remains a subject of debate.

Conventional oxygen therapy (COT) via nasal cannulas or face masks is often used for maintaining oxygenation during FB. However, COT is less efficient in patients with moderate to severe respiratory impairment. The low flow rates achievable with COT may be insufficient to maintain adequate oxygenation in the presence of hypoventilation induced by sedative medications, pre-existing pulmonary dysfunction, or prolonged procedural duration. Furthermore, airway sharing inherent to flexible bronchoscopy may interfere with optimal oxygen delivery and reduce the ability to promptly correct hypoxemic episodes, regardless of the oxygen delivery device used [10,11].

Non-invasive ventilation (NIV) can overcome some of the limitations of COT. Several studies have demonstrated that NIV can reduce the frequency and severity of desaturation during FB, maintain adequate gas exchange, and decrease the need for post-procedure invasive ventilatory support [12,13].

NIV has been particularly effective in patients with significant hypoxemia or respiratory compromise, not only in maintaining ventilation, but also conferring greater hemodynamic stability during procedures [14]. The British Thoracic Society and other international guidelines recommend the use of NIV in hypoxemic patients undergoing FB in settings where facilities for emergent intubation and advanced ventilatory support are immediately available [15]. However, NIV is rarely used because of patients’ intolerance and difficulty advancing the bronchoscope through the nares, and patient-ventilator asynchrony can occur, adding to discomfort and complicating the procedure.

High-flow nasal cannula (HFNC) has recently emerged as a novel solution for non-invasive oxygen delivery, offering several physiological advantages over both COT and NIV. HFNC delivers heated and humidified oxygen at high flow rates, allowing for the administration of higher inspired oxygen fractions and generating a low level of positive end-expiratory pressure (PEEP), typically 2–5 cm H_2_O depending on flow rate and whether the mouth is closed, which increases end-expiratory lung volume and thus increases functional residual capacity (FRC). HFNC also reduces dead space by flushing the nasopharyngeal and upper airway during expiration, thereby decreasing rebreathing of CO_2_ and improving ventilatory efficiency. This dead space washout effect is flow-dependent and is particularly beneficial during procedures like bronchoscopy, where airway instrumentation can further compromise ventilation. The reduction in dead space leads to lower PaCO_2_ and less work of breathing, as fresh oxygen-rich gas is available at the start of each inspiration [16,17]. These effects help maintain alveolar recruitment, prevent atelectasis, and improve ventilation-perfusion matching, leading to better oxygenation during bronchoscopy.

In addition, HFNC enhances mucociliary clearance, reduces airway resistance and bronchoconstriction, and is generally well tolerated by patients, even during prolonged procedures [18]. These properties make HFNC a particularly attractive option for patients with moderate respiratory dysfunction, in whom the risks of hypoxemia and hypercapnia are increased [19,20].

Several studies have evaluated the role of HFNC in maintaining adequate oxygenation and ventilation during bronchoscopic procedures, demonstrating a reduction in incidence of procedure-related desaturation, minimized need for endotracheal intubation, and enhanced patient comfort compared to conventional oxygen therapy [21,22,23,24]. Available research is still sparse on the use of HFNC specifically in patients with moderate respiratory dysfunction—a population that is particularly vulnerable to procedural complications yet may benefit most from optimized non-invasive oxygen delivery strategies.

Given this context, the present study aims to assess the feasibility, safety, and clinical impact of HFNC in patients with moderate respiratory impairment undergoing FB. Specifically, we sought to evaluate respiratory and hemodynamic parameters, sedation tolerance, and the incidence of procedure-related adverse events, with the overarching goal of determining whether HFNC can serve as a reliable and effective oxygenation strategy in this high-risk patient population. By providing detailed clinical observations, this study aims to contribute to the growing body of evidence supporting the use of HFNC as a safe and efficacious modality for respiratory support during invasive airway procedures.

## 2. Materials and Methods

### 2.1. Study Design and Setting

This retrospective, single-center observational study was conducted between January and May 2025 following the introduction of high-flow nasal cannula (HFNC) oxygen therapy into routine clinical practice, in accordance with institutional protocols.

### 2.2. Study Population

Adult patients (≥18 years) with a body mass index (BMI) between 18 and 30 kg/m^2^ were eligible for inclusion. Patients were required to have moderate respiratory dysfunction, defined by at least one of the following criteria: peripheral oxygen saturation (SpO_2_) between 88% and 92% on room air; arterial blood gas (ABG) analysis showing partial pressure of oxygen (PaO_2_) between 60 and 70 mmHg; and/or partial pressure of carbon dioxide (PaCO_2_) between 45 and 55 mmHg, with a PaO_2_/FiO_2_ (P/F) ratio between 100 and 200. Pulmonary function tests (PFTs) were required to demonstrate forced expiratory volume in 1 s (FEV_1_) between 50% and 70% of predicted, forced vital capacity (FVC) between 50% and 80% of predicted, and diffusing capacity of the lung for carbon monoxide (DLCO) reduced by 40–60%.

Moderate respiratory failure was classified according to established clinical criteria, consistent with previously published definitions referenced in the Discussion.

Exclusion criteria included intolerance to or contraindications for HFNC therapy (e.g., recent facial trauma or esophageal surgery) and the requirement for invasive mechanical ventilation.

### 2.3. Bronchoscopy Procedure and Sedation

Flexible bronchoscopy (FB) was performed via the oral route by an experienced operator. Continuous monitoring was maintained throughout the procedure, including non-invasive blood pressure, three-lead electrocardiography (ECG), SpO_2_, and end-tidal carbon dioxide (ETCO_2_).

Sedation was administered by an anesthesiologist using intravenous midazolam and/or propofol, targeting a Richmond Agitation–Sedation Scale (RASS) score of −2 to −3 while preserving spontaneous breathing. Supplemental analgesics were not routinely used. To minimize the risk of respiratory depression, sedative agents were titrated incrementally under continuous respiratory and hemodynamic monitoring.

### 2.4. Data Collection

Collected variables included demographic data (age, sex, BMI), baseline pulmonary function test results, and pre-procedural respiratory parameters (SpO_2_, PaO_2_, and PaCO_2_ from ABG analysis). Procedural data comprised bronchoscopy duration, hemodynamic variables, and cumulative sedative doses.

All patients received HFNC oxygen therapy (AIRVO™ 3, Fisher & Paykel Healthcare Irvine, CA, USA) with a flow rate of 60 L/min and a fraction of inspired oxygen (FiO_2_) between 40% and 60%, adjusted to maintain SpO_2_ > 92%.

### 2.5. Outcomes

The primary outcome was the occurrence of severe hypoxemia, defined as a decrease in SpO_2_ below 90%. Secondary outcomes included the need for airway rescue maneuvers (jaw thrust or chin lift), conversion to alternative ventilatory support (including endotracheal intubation for mechanical ventilation), and procedural interruption.

### 2.6. Bias and Sample Size

Potential sources of bias included selection bias due to the absence of a comparator group, confounding bias related to the non-randomized design, and limited external validity owing to the single-center setting. The sample size was determined by the inclusion of consecutive eligible patients during the study period.

### 2.7. Statistical Analysis

Statistical analysis was descriptive in nature. Continuous variables are presented as mean ± standard deviation (SD) and median values. Given the small sample size, 95% confidence intervals (CIs) for continuous variables were calculated using Student's *t* distribution. Categorical variables are reported as absolute numbers and percentages, with corresponding 95% confidence intervals for proportions. No formal hypothesis testing was planned, and *p* values were not considered a primary measure of effect. All analyses were performed using Microsoft Excel.

## 3. Results

Patient characteristics are summarized in Table 1. Twenty consecutive patients undergoing flexible bronchoscopy were included in the analysis. The cohort was characterized by advanced age and impaired respiratory function. The mean age was 67 ± 8 years (95% CI 64.3–71.7), and 12 patients (60%; 95% CI 36–81) were female. The mean body mass index was 25 ± 3 kg/m^2^ (95% CI 23.1–26.1). At baseline, mean peripheral oxygen saturation was 94 ± 3% (95% CI 92.3–95.1). Arterial blood gas analysis showed a mean PaO_2_ of 74 ± 9 mmHg (95% CI 69.5–77.5), a mean PaCO_2_ of 48 ± 9 mmHg (95% CI 43.6–52.0), and a mean PaO_2_/FiO_2_ ratio of 162 ± 19 (95% CI 153.1–170.9). Pulmonary function tests demonstrated moderate impairment, with a mean FEV1 of 69 ± 9% predicted (95% CI 47.3–59.1), mean FVC of 75 ± 6% predicted (95% CI 72.7–78.1), and mean DLCO of 66 ± 14% predicted (95% CI 60.2–73.0).

A summary of procedural findings and the outcome studied is shown in Table 2.

Flexible bronchoscopy was successfully completed in all patients. The mean duration of the procedure was 9 ± 2 min (95% CI 8.1–9.9). The mean oxygen flow delivered during the procedure was 53 ± 9 L/min (95% CI 48.8–57.2).

Sedation consisted of a fixed low dose of midazolam (median 3 mg) combined with propofol, with a mean propofol dose of 8.5 ± 9.3 mg (95% CI 4.1–12.9).

### Safety Outcomes

No clinically relevant adverse respiratory or hemodynamic events were observed. Namely, no episodes of desaturation occurred during bronchoscopy (0/20 patients; 0%, 95% CI 0–15), and oxygen saturation remained consistently high throughout the procedure. The lowest recorded SpO_2_ was 98.6% (95% CI 98.2–99.0), while the highest recorded value was 99.85% (95% CI 99.7–100). All procedures were completed without interruption, and no patient required invasive ventilation (0/20; 95% CI 0–15). Furthermore, no airway support maneuvers, such as jaw thrust or chin lift, were required, and no episodes of intra-procedural hypotension (mean arterial pressure < 65 mmHg) were recorded.

## 4. Discussion

This study provides insights into the safety and feasibility of HFNC in patients with moderate respiratory deficiency undergoing FB. The mean procedural duration was approximately 9 min, reflecting efficient execution within standard clinical practice. Sedation protocols using low to moderate doses of midazolam and propofol allowed maintenance of adequate sedation levels, with a RASS score between −2/−3, while preserving spontaneous ventilation, as evidenced by the absence of any desaturation episodes (SpO_2_ < 90%) during all procedures.

Importantly, the highest and lowest oxygen saturation values remained stable and within safe limits. No patients required procedural interruption, escalation to invasive mechanical ventilation, or airway rescue maneuvers such as jaw thrust or chin lift. Additionally, there were no recorded episodes of severe hypotension, indicating hemodynamic stability throughout the procedures.

Current clinical practice guidelines recommend providing supplemental oxygen to prevent hypoxemia during flexible bronchoscopy. From a cost–benefit perspective, it is important to tailor respiratory support strategies to the patient’s individual risk profile. Luo et al. [25] demonstrated that patients with mild hypoxemia and P:F ratios greater than 200 derived only limited benefit from HFNC compared with COT, whereas those with lower P:F ratios experienced a substantial reduction in hypoxemic events when managed with HFNC. These findings align with the results of Kaya et al. [26], who similarly identified a low P:F ratio as a risk factor for intraprocedural hypoxia. The authors also suggested that varying indications for bronchoscopy may differentially influence the risk of procedural hypoxia, warranting further investigation.

In this context, some authors have questioned the routine use of oxygen supplementation in patients with only mild respiratory impairment, whereas others have shown that the risk of desaturation becomes significant primarily in individuals with markedly reduced FEV_1_ [27]. More recently, Choi et al. [28] screened a cohort of 2520 patients undergoing FB under sedation and found a significant association between procedural desaturations and age older than 60, low FEV_1_ and duration over 40 min. In accordance with their findings, patients in our study were at greater risk due to their age (average 67) and low FEV_1_ (on average 69), while the average duration of 9 min might have represented a protective factor against hypoxemia. Smoother execution without interruptions and adequate level of sedation might have contributed to shorter duration, possibly relieving the cohort from the risk for hypoxia. Ben Menachem et al. [29] demonstrated how HFNC can dramatically reduce procedure duration, especially in high-risk patients, as lung transplant recipients. Other authors [30,31] also demonstrated a lower rate of rescue interventions as jaw thrust and chin lift when comparing HFNC versus standard bag mask ventilation, in accordance with our results.

In line with existing evidence, our study indicates that patients with moderate respiratory dysfunction represent the subgroup most likely to benefit from this ventilatory strategy, whereas patients with mild impairment may achieve comparable outcomes with conventional oxygen therapy, and those with severe respiratory dysfunction may not obtain adequate ventilatory support from HFNC alone.

Despite positive results reported in several studies, the use of high-flow nasal cannula (HFNC) during flexible bronchoscopy (FB) still requires further investigation to optimize its implementation. Multiple studies have shown that HFNC reduces hypoxemic events in both diagnostic and therapeutic procedures. For example, Longhini et al. [32] evaluated HFNC during bronchoalveolar lavage in patients with suspected pneumonia and reported fewer hypoxemic episodes and a favorable safety profile. These findings emphasize the need to tailor oxygen therapy according to the severity and type of underlying lung disease when performing FB.

Supporting this, Roy et al. [33] conducted a systematic review and meta-analysis comparing HFNC with conventional oxygen therapy (COT) during bronchoscopy. The use of HFNC during bronchoscopy was associated with a reduction in the frequency of desaturation events [relative risk (RR) 0.34, 95% (CI) 0.27–0.44, I^2^ = 23%], a higher lowest SpO_2_ value [mean difference (MD) 4.30, 95% CI 2.41–6.19, I^2^ = 96%], and an increase in PaO_2_ from baseline (MD 21.77, 95% CI 2.8–40.74, I^2^ = 99%), while PaCO_2_ levels remained comparable (MD −0.34, 95% CI −1.82 to 1.13, I^2^ = 58%) immediately post-procedure. In an RCT performed by Douglas et al. [22] oxygen saturation following pre-oxygenation and the lowest oxygen saturation recorded during bronchoscopy were markedly higher in the high-flow nasal oxygen group compared to the standard oxygen therapy group: median (IQR [range]) 100 (99–100 [93–100]) vs. 98 (97–99 [94–100]), *p* = 0.0001, and 97.5 (94–99 [77–100]) vs. 92 (88–95 [79–98]), *p* < 0.001, respectively. The heterogeneity in pulmonary disease severity among study populations may influence CO_2_ clearance and represent a potential source of bias.

The anatomical advantages of HFNC further facilitate bronchoscopy. Its nasal interface allows patients to keep their mouths mostly closed during the procedure, maintaining oxygen delivery, positive end-expiratory pressure, and CO_2_ removal [34]. Historically, some authors have explored prophylactic or intraoperative insertion of nasopharyngeal tubes to manage hypoxia during FB [35,36]; while effective, invasive ventilation carries notable cardiovascular and respiratory risks and may not be justified for relatively short procedures.

NIV has also been used as an adjunctive support during FB in critically ill patients to prevent adverse events and reduce the need for endotracheal intubation [37]. NIV allows high mean airway pressures and inspiratory flows and simple triggering, which can facilitate bronchoscopy. However, concerns exist regarding side effects such as gastric distension [38] due to oral insertion of the bronchoscope and the increased risk of gas aspiration compared with HFNC. Saksitthichok et al. [39] reported greater post-procedural dyspnea with NIV, and Simon et al. [40] found that while NIV improved oxygenation compared to HFNC in patients with acute hypoxemic respiratory failure, it was also associated with higher reintubation rates within 24 h. Our study was mainly focused on procedural outcomes, and we did not include a 24-h follow-up period, which may represent a limitation.

HFNC offers several advantages over NIV and COT, including ease of use, delivery of heated, humidified flows up to 60 L/min, and provision of end-expiratory positive pressure (~0.7 cm H_2_O per 10 L/min) [24]. It effectively reduces hypoxemic episodes and lowers intubation requirements [41]. Compared with tight-fitting NIV masks, HFNC provides greater patient comfort, alleviates dyspnea, and can be applied in various clinical settings, including dedicated bronchoscopy suites [42]. Recent reviews indicate that HFNC outperforms COT in patients with moderate respiratory impairment, while NIV may be reserved for those with more severe acute respiratory failure [43].

Several studies have further examined the optimization of HFNC settings. Service et al. [44] reported that HFNC reduced desaturation rates and received positive feedback from clinicians, although optimal flow rates remain to be established. Lucangelo et al. [45] compared Venturi Mask with HFNC at 40 and 60 L/min, demonstrating that higher flow rates improved outcomes, likely due to increased PEEP and airway patency. Similarly, Zhang and colleagues [31] identified an optimal flow of 50–60 L/min to prevent desaturations, consistent with the average flow used in our study (53 L/min).

While previous studies have yielded mixed evidence regarding the influence of comorbidities and baseline severity on bronchoscopy-related complications, with some authors reporting no association [28] and others highlighting substantial effects on desaturation rates [26], and post-procedural intubation, and overall complication burden, our ability to draw similarly robust conclusions is constrained by the methodological limitations of the present study. Specifically, the small sample size reduces statistical power and may obscure infrequent adverse events or subtle differences across patient subgroups. Additionally, the observational, single-center design introduces potential selection bias and limits generalizability. Heterogeneity in underlying respiratory conditions, sedation requirements, and indications for bronchoscopy further increases the risk of confounding, complicating efforts to disentangle the true impact of baseline patient characteristics on clinical outcomes [46].

Despite the small sample size and the resulting limitations in external validity, this preliminary evaluation suggests that the procedure is safe and effective, characterized by ease of implementation and low sedative requirements. These findings support the potential applicability of HFNC during bronchoscopy in tertiary care centers, including settings with limited resources.

The lack of a control group, such as patients managed with conventional oxygen therapy, limits the ability to draw comparative conclusions regarding the effectiveness of HFNC. Future studies with controlled or randomized designs, including appropriate comparison groups, are needed to validate these preliminary observations.

Future study designs should accurately stratify patient populations with the scope of evaluating immediate procedural outcomes but also delayed complications and longer-term effects of HFNC. Cost-effectiveness analysis could be included in future studies comparing different patient subgroups, possibly structured as RCTs with multiple arms, comparing HFNC to other ventilatory strategies. Additional research is needed to investigate in detail blood exchange dynamics in diverse patient populations undergoing bronchoscopic procedures, and larger-scale studies to confirm these promising results.

## 5. Conclusions

HFNC can be a simple and effective strategy to support ventilation in patients with moderate respiratory dysfunction during FB, facilitating completion of the procedure without severe desaturations and other adverse events. Among patients with varying degrees of respiratory impairment, those with moderate respiratory dysfunction appear to represent the subgroup most likely to derive benefit from this ventilatory strategy.

## Figures and Tables

**Table 1:** Demographic and Clinical Characteristics of Patients (*n* = 20).

	Mean ± SD/*n* (%)	Range
Age (years)	67.0 ± 8	56–82
Sex (M/F)	9 (45%)/11 (55%)	—
BMI (kg/m^2^)	25 ± 3	20–30
Baseline SpO_2_ (%)	94 ± 3	88–97
PaO_2_ (mmHg)	74 ± 9	60–87
PaCO_2_ (mmHg)	48 ± 9	31–59
P/F ratio	162 ± 19	136–200
FEV_1_ (% predicted)	69 ± 9	50–80
FVC (% predicted)	75 ± 6	65–82
DLCO (% predicted)	66 ± 14	40–85

Abbreviations: BMI, Body Mass Index; FEV_1_, Forced Expiratory Volume in 1 min; FVC, Forced Vital Capacity; DLCO, Diffusing Capacity of the Lungs for Carbon Dioxide.

**Table 2:** Procedural safety and oxygenation outcomes during flexible bronchoscopy with HFNC support (*n* = 20).

	Mean ± SD/*n* (%)	Range
Duration of FB (min)	9 ± 2	6–13
Flow (L/min)	53 ± 9 *	40–60
Total midazolam dose (mg)	3.0 ± 0.8	2–5
Total propofol dose (mg)	8.5 ± 9.3	0–20
Occurrence of desaturation (SpO_2_ < 90%)	0 (0%)	—
Highest SpO_2_ during FB (%)	99.9 ± 0.4	99–100
Lowest SpO_2_ during FB (%)	98.6 ± 0.8	97–99
Procedural interruption	0 (0%)	—
Conversion to invasive ventilation	0 (0%)	—
Jaw thrust/chin lift required	0 (0%)	—
Severe hypotension	0 (0%)	—

* Median 60. The values are represented as means and standard deviations.

## Data Availability

The corresponding author can provide databases and literature screening upon valid request.

## Abbreviations

The following abbreviations are used in this manuscript:

FB	Flexible bronchoscopy
NIV	Noninvasive ventilation
HFNC	High-flow nasal cannula
PFT	Pulmonary Functional Test
ABG	Arterial Blood Gas
BAL	Bronchoalveolar lavage
COT	Conventional oxygen therapy
RASS	Richmond Agitation Sedation Scale

## References

[1] Miller R.J., Casal R.F., Lazarus D.R., Ost D.E., Eapen G.A. (2018). Flexible Bronchoscopy. Clin. Chest Med..

[2] Matthes S., Treml M., Hübner R.H., Hetzel J., Eberhardt R., Franke K.J., Herth F.J., Holland A., Loop T., Sitter H. (2025). A Systematic Review of the Safety of Sedation during Flexible Bronchoscopy. Respiration.

[3] Wang L., Wang M., Xu R., Li J., Cao Y., Ji F. (2025). Comparative analysis of guidelines and recommendations for sedation during flexible bronchoscopy: A narrative review. Eur. Respir. Rev..

[4] Montravers P., Gauzit R., Dombret M.C., Blanchet F., Desmonts J.M. (1993). Cardiopulmonary effects of bronchoalveolar lavage in critically ill patients. Chest.

[5] Pirożyński M., ⋅Sliwiński P., Radwan L., Zieliński J. (1991). Bronchoalveolar Lavage: Comparison of Three Commonly Used Procedures. Respiration.

[6] Davies L., Mister R., Spence D.P., Calverley P.M., Earis J.E., Pearson M.G. (1997). Cardiovascular consequences of fibreoptic bronchoscopy. Eur. Respir. J..

[7] Katz A.S., Michelson E.L., Stawicki J., Holford F.D. (1981). Cardiac arrhythmias. Frequency during fiberoptic bronchoscopy and correlation with hypoxemia. Arch. Intern. Med..

[8] D’Ippolito R., Foresi A., Castagnetti C., Gesualdi S., Castagnaro A., Marangio E., Olivieri D. (2007). Indications for flexible fiberoptic bronchoscopy and its safety in the very elderly. Monaldi Arch. Chest Dis..

[9] Saito Z., Oi I., Ito T., Imakita T., Kanai O., Fujita K., Tachibana H., Mio T. (2023). Safety and Efficacy of Flexible Bronchoscopy in Elderly Patients: A Retrospective Comparative Study. Open Respir. Arch..

[10] Sheahan C.G., Mathews D.M. (2014). Monitoring and delivery of sedation. Br. J. Anaesth..

[11] Costa R., Navalesi P., Cammarota G., Longhini F., Spinazzola G., Cipriani F., Ferrone G., Festa O., Antonelli M., Conti G. (2017). Remifentanil effects on respiratory drive and timing during pressure support ventilation and neurally adjusted ventilatory assist. Respir. Physiol. Neurobiol..

[12] Hidalgo Sánchez M., Luján M., Alcolea Batres S., Álvarez Del Vayo J., Mariscal-Aguilar P., Carpio C., Álvarez-Sala Walther R. (2025). Evidence on Non-Invasive Respiratory Support During Flexible Bronchoscopy: A Narrative Review. J. Clin. Med..

[13] Nong L., Liang W., Yu Y., Xi Y., Liu D., Zhang J., Zhou J., Yang C., He W., Liu X. (2020). Noninvasive ventilation support during fiberoptic bronchoscopy-guided nasotracheal intubation effectively prevents severe hypoxemia. J. Crit. Care.

[14] Antonelli M., Conti G., Rocco M., Arcangeli A., Cavaliere F., Proietti R., Meduri G.U. (2002). Noninvasive Positive-Pressure Ventilation vs Conventional Oxygen Supplementation in Hypoxemic Patients Undergoing Diagnostic Bronchoscopy. Chest.

[15] Du Rand I.A., Blaikley J., Booton R., Chaudhuri N., Gupta V., Khalid S., Mandal S., Martin J., Mills J., Navani N. (2013). British Thoracic Society guideline for diagnostic flexible bronchoscopy in adults: Accredited by NICE. Thorax.

[16] Drake M.G. (2018). High-Flow Nasal Cannula Oxygen in Adults: An Evidence-based Assessment. Ann. Am. Thorac. Soc..

[17] Papazian L., Corley A., Hess D., Fraser J.F., Frat J.P., Guitton C., Jaber S., Maggiore S.M., Nava S., Rello J. (2016). Use of high-flow nasal cannula oxygenation in ICU adults: A narrative review. Intensive Care Med..

[18] Möller W., Feng S., Domanski U., Franke K.J., Celik G., Bartenstein P., Becker S., Meyer G., Schmid O., Eickelberg O. (2017). Nasal high flow reduces dead space. J. Appl. Physiol..

[19] Wei C., Ma S., Wang J., Yang N., Wang D., Yuan L., Wang Y. (2024). The effectiveness of transnasal high flow nasal cannula in bronchoscopy under sedation: A systematic review and meta-analysis. Front. Med..

[20] Mauri T., Turrini C., Eronia N., Grasselli G., Volta C.A., Bellani G., Pesenti A. (2017). Physiologic Effects of High-Flow Nasal Cannula in Acute Hypoxemic Respiratory Failure. Am. J. Respir. Crit. Care Med..

[21] Chung S.M., Choi J.W., Lee Y.S., Choi J.H., Oh J.Y., Min K.H., Hur G.Y., Lee S.Y., Shim J.J., Kang K.H. (2019). Clinical Effectiveness of High-Flow Nasal Cannula in Hypoxaemic Patients during Bronchoscopic Procedures. Tuberc. Respir. Dis..

[22] Douglas N., Ng I., Nazeem F., Lee K., Mezzavia P., Krieser R., Steinfort D., Irving L., Segal R. (2018). A randomised controlled trial comparing high-flow nasal oxygen with standard management for conscious sedation during bronchoscopy. Anaesthesia.

[23] Irfan M., Ahmed M., Breen D. (2021). Assessment of High Flow Nasal Cannula Oxygenation in Endobronchial Ultrasound Bronchoscopy. J. Bronchol. Interv. Pulmonol..

[24] Wang R., Li H.C., Li X.Y., Tang X., Chu H.W., Yuan X., Tong Z.H., Sun B. (2021). Modified high-flow nasal cannula oxygen therapy versus conventional oxygen therapy in patients undergoing bronchoscopy: A randomized clinical trial. BMC Pulm. Med..

[25] Luo X., Xiang F. (2024). High-flow nasal cannula oxygen therapy versus conventional oxygen therapy in patients undergoing bronchoscopy: A retrospective study. BMC Pulm. Med..

[26] Gürün Kaya A., Öz M., Dilegelen U., Ecer D., Erol S., Çiftçi F., Çiledağ A., Kaya A. (2023). Is Flexible Bronchoscopy a Safe Procedure for Critical Care Patients with Respiratory Failure?. Acta Clin. Croat..

[27] Jones A.M., O'Driscoll R. (2001). Do all patients require supplemental oxygen during flexible bronchoscopy?. Chest.

[28] Choi J.S., Lee E.H., Lee S.H., Leem A.Y., Chung K.S., Kim S.Y., Jung J.Y., Kang Y.A., Park M.S., Chang J. (2020). Risk Factors for Predicting Hypoxia in Adult Patients Undergoing Bronchoscopy under Sedation. Tuberc. Respir. Dis..

[29] Ben-Menachem E., McKenzie J., O’Sullivan C., Havryk A.P. (2020). High-flow Nasal Oxygen Versus Standard Oxygen During Flexible Bronchoscopy in Lung Transplant Patients. J. Bronchol. Interv. Pulmonol..

[30] Wu C.N., Xia L.Z., Li K.H., Ma W.H., Yu D.N., Qu B., Li B.X., Cao Y. (2020). High-flow nasal-oxygenation-assisted fibreoptic tracheal intubation in critically ill patients with COVID-19 pneumonia: A prospective randomised controlled trial. Br. J. Anaesth..

[31] Zhang W., Yuan X., Shen Y., Wang J., Xie K., Chen X. (2024). Optimal flow of high-flow nasal cannula oxygenation to prevent desaturation during sedation for bronchoscopy: A randomized controlled study. Ther. Adv. Respir. Dis..

[32] Longhini F., Pelaia C., Garofalo E., Bruni A., Placida R., Iaquinta C., Arrighi E., Perri G., Procopio G., Cancelliere A. (2022). High-flow nasal cannula oxygen therapy for outpatients undergoing flexible bronchoscopy: A randomised controlled trial. Thorax.

[33] Roy A., Khanna P., Chowdhury S.R., Haritha D., Sarkar S. (2022). The Impact of High-flow Nasal Cannula vs Other Oxygen Delivery Devices during Bronchoscopy under Sedation: A Systematic Review and Meta-analyses. Indian J. Crit. Care Med..

[34] de Boer G.M., Türk Y., Waning V.H.M.-V., Braunstahl G.-J. (2017). Bronchoscopy: Oral or Nasal Insertion?. J. Bronchol. Interv. Pulmonol..

[35] Chhajed P.N., Glanville A.R. (2003). Management of hypoxemia during flexible bronchoscopy. Clin. Chest Med..

[36] Chhajed P.N., Aboyoun C., Malouf M.A., Hopkins P.M., Plit M., Grunstein R.R., Glanville A.R. (2003). Prophylactic nasopharyngeal tube insertion prevents acute hypoxaemia due to upper-airway obstruction during flexible bronchoscopy. Intern. Med. J..

[37] Huang W., Wu F., Tian L., Xu C., Wang H. (2024). Clinical practice guidelines in adult diagnostic flexible bronchoscopy: Systematic review of the literature and quality appraisal with AGREE II. J. Thorac. Dis..

[38] Ozyilmaz E., Ugurlu A.O., Nava S. (2014). Timing of noninvasive ventilation failure: Causes, risk factors, and potential remedies. BMC Pulm. Med..

[39] Saksitthichok B., Petnak T., So-ngern A., Boonsarngsuk V. (2019). A prospective randomized comparative study of high-flow nasal cannula oxygen and non-invasive ventilation in hypoxemic patients undergoing diagnostic flexible bronchoscopy. J. Thorac. Dis..

[40] Simon M., Braune S., Frings D., Wiontzek A.K., Klose H., Kluge S. (2014). High-flow nasal cannula oxygen versus non-invasive ventilation in patients with acute hypoxaemic respiratory failure undergoing flexible bronchoscopy—A prospective randomised trial. Crit. Care.

[41] Jackson J.A., Spilman S.K., Kingery L.K., Oetting T.W., Taylor M.J., Pruett W.M., Omerza C.R., Branick K.A., Ganapathiraju I., Hamilton M.Y. (2021). Implementation of High-Flow Nasal Cannula Therapy Outside the Intensive Care Setting. Respir. Care.

[42] Zemach S., Helviz Y., Shitrit M., Friedman R., Levin P.D. (2019). The Use of High-Flow Nasal Cannula Oxygen Outside the ICU. Respir. Care.

[43] Pelaia C., Bruni A., Garofalo E., Rovida S., Arrighi E., Cammarota G., Navalesi P., Pelaia G., Longhini F. (2021). Oxygenation strategies during flexible bronchoscopy: A review of the literature. Respir. Res..

[44] Service J.A., Bain J.S., Gardner C.P., McNarry A.F. (2019). Prospective Experience of High-flow Nasal Oxygen During Bronchoscopy in 182 Patients. J. Bronchol. Interv. Pulmonol..

[45] Lucangelo U., Vassallo F.G., Marras E., Ferluga M., Beziza E., Comuzzi L., Berlot G., Zin W.A. (2012). High-flow nasal interface improves oxygenation in patients undergoing bronchoscopy. Crit. Care Res. Pract..

[46] Li J., Jing G., Scott J.B. (2020). Year in Review 2019: High-Flow Nasal Cannula Oxygen Therapy for Adult Subjects. Respir. Care.

